# Roar: detecting alternative polyadenylation with standard mRNA sequencing libraries

**DOI:** 10.1186/s12859-016-1254-8

**Published:** 2016-10-18

**Authors:** Elena Grassi, Elisa Mariella, Antonio Lembo, Ivan Molineris, Paolo Provero

**Affiliations:** 1 Department of Molecular Biotechnology and Health Sciences, Molecular Biotechnology Center, Via Nizza 52, Torino, 10126 Italy; 2 Center for Translational Genomics and Bioinformatics, San Raffaele Scientific Institute, Via Olgettina 60, Milan, 20132 Italy

**Keywords:** 3’ UTR, Polyadenylation, RNA-sequencing, Software, Bioconductor

## Abstract

**Background:**

Post-transcriptional regulation is a complex mechanism that plays a central role in defining multiple cellular identities starting from a common genome. Modifications in the length of 3’UTRs have been found to play an important role in this context, since alternative 3’ UTRs could lead to differences for example in regulation by microRNAs and cellular localization of the transcripts thus altering their fate.

**Results:**

We propose a strategy to identify the genes undergoing regulation of 3’ UTR length using RNA sequencing data obtained from standard libraries, thus widely applicable to data originally obtained to perform classical differential expression analyses. We decided to exploit previously annotated APA sites from public databases, in contrast with other approaches recently proposed in which the location of the APA site is inferred from the data together with the relative abundance of the isoforms.

We demonstrate the reliability of our method by comparing it to the results of other microarray based or specific RNA-seq libraries methods and show that using APA sites databases results in higher sensitivity compared to de novo site prediction approach.

**Conclusions:**

We implemented the algorithm in a Bioconductor package to facilitate its broad usage in the scientific community. The ability of this approach to detect shortening from libraries with a number of reads comparable to that needed for differential expression analyses makes it useful for investigating if alternative polyadenylation is relevant in a certain biological process without requiring specific experimental assays.

**Electronic supplementary material:**

The online version of this article (doi:10.1186/s12859-016-1254-8) contains supplementary material, which is available to authorized users.

## Background

Gene regulation is a complex set of mechanisms used by living organisms to generate different cell types and behaviors from a single genome. Among the most recent discoveries in this field is the dynamic and highly polymorphic nature of 3’ Untranslated Regions (3’ UTRs) [1–3]. These regions play a fundamental role in regulating transcript abundance, translation and localization, and have recently been shown to be highly polymorphic both among tissues [4, 5] and individuals [6]. Most human genes have multiple alternative polyadenylation (APA) sites and thus are able to give rise to primary transcripts with different 3’ ends. The most common APA form consists in the cleavage of the transcript in a position that is more 5’ proximal than the canonical one but still in the 3’UTR, thus leaving the coding sequence unmodified but leading to a “shortened” processed mRNA.

APA can have multiple effects on the fate of the transcripts, since 3’UTRs harbor recognition sites for microRNAs and several RNA binding protein affecting transcripts stability and cellular localization; moreover also nuclear export [7] and translational efficiency are influenced by 3’UTRs [3].

Some general trends have been identified in recent studies of APA: cells in highly proliferative normal tissues (i.e. testes) and cancer cells express a higher number of shortened transcripts than non-proliferating tissues (i.e. brain) and the healthy counterparts of tumors [3, 8, 9]. In agreement with these observations a trend towards longer 3’ UTRs has been identified during murine embryonic differentiation [10].

The approaches that have been proposed so far to study this process on a genomic level were based on specific procedures to process microarray signals [11] while in the last years many ad hoc RNA-sequencing protocols were developed in order to identify the location of APA sites in transcripts, and the expression levels of different isoforms in different contexts [12].

Given the high number of already available RNAseq data obtained for differential expression analyses we sought to develop a software able to identify differential APA sites usage across different conditions on these datasets, without requiring ad hoc sequencing approaches able to detect specifically the ends of transcripts (i.e. SAPAS by [13]).

Recently the APA phenomenon has been studied in several normal tissues and species with ad hoc sequencing approaches making available the coordinates of many of the possible ends of transcripts in public databases (PolyA_DB2 [14] and APASdb [15]), therefore we decided to exploit these already annotated APA sites to identify the alternative transcripts whose expression levels we want to compare. This choice should be contrasted with other approaches recently proposed [16–23], in which the location of the APA site is inferred from the data together with the relative abundance of the isoforms. The advantage of our choice is, as shown in the results, higher sensitivity in detecting alternative usage of annotated APAs compared to a tool that infers APA location from RNA-seq data.

Using RNAseq and APA databases to distinguish different isoform expression and specifically alternative polyadenylation is not a novel idea: [24] used ratio of RNAseq read density or average microarray probe intensity on different portions of UTRs to define the Relative expression of mRNA isoforms Using Distal polyA sites - here we extend their RNAseq based approach to compare the polyadenylation status between two different conditions in a statistically robust manner and propose a Bioconductor package to make differential APA analyses an easily added step to every mRNAseq experiment.

## Implementation

Our approach is based on defining two distinct portions of the ends of the transcripts: one shared by both the short and long isoforms, which from now on will be addressed as PRE, and the other one (POST) that pertains only to the long isoforms. Using reads falling on these two regions for a given gene we are able to obtain the expression ratio (m/M) between the short and long isoforms in a given sample. To compare different conditions we calculate the ratio of the two m/M obtained in different samples: this Ratio Of A Ratio is called roar and represents the tendency of the first condition to express relatively higher levels of the short isoform (when roar >1) or higher levels of the long one (when roar <1).

To evaluate the statistical significance of such difference we use a Fisher test, following [25], comparing the imbalance between the PRE and POST read counts in the two conditions. When there is more than one sample for each condition the roar calculations are performed on mean read counts; then if the experimental design is unpaired all the possible sample combinations are evaluated with the Fisher test, otherwise only tests comparing paired samples are performed and then their *p*-values are combined using the Fisher method ([26]).

In the analyses presented here we identify shortened and lengthened genes according to these criteria:

1. cutoff on the expression levels of the gene (in both conditions):
  $$FPKM_{PRE} > 1 $$
2. a roar value >1 (shortening) or <1 (lengthening). Note that negative or undefined values of m/M or roar could occur in some situations - such as counts equal to zero for PRE or POST portions - and are discarded
3. the Bonferroni corrected^1^ Fisher test *p*-value <0.05 for single samples analyses, while for multiple samples cases we require that all samples crossings result in a nominal *p*-value <0.05

*F* *P* *K* *M*_*PRE*_ is simply the FPKM value obtained for the PRE fragment, reflecting the abundance of the given gene.

### Definition of PRE and POST portions

**Fig. 1:**
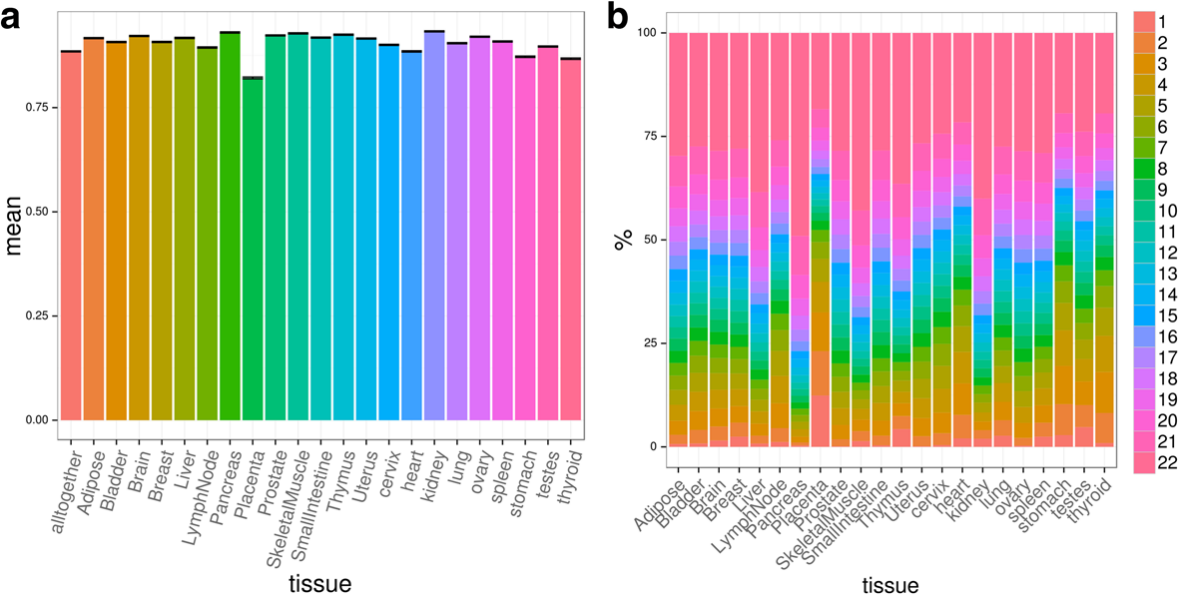
APASdb reported sites across tissues. **a** - mean (+/- sem) of the fraction of reads assigned to the two sites with more reads for every gene with at least two overlapping sites in APASdb across different tissues. For “alltogether” we put together sites annotations for all the 22 human normal tissues, normalizing reads with respect to the total number of reads found in that tissue and considering the sites supported by more than 2 normalized reads. **b** - percentages of sites found in different tissues that are found in other N tissues: on average 29 % of sites are found in all tissues. 50 % of the sites are found in at least 17 tissues

Our approach could be used for genes with either a single polyadenylation site or multiple ones, but as long as in the majority of cases even genes with many reported polyadenylation sites predominantly use only two of them (0.9 is the average ratio of reads supporting the two most used sites in APASdb over total reads for the sites falling over a gene, see Fig. 1), we offer a simplified procedure that deals with a univocal PRE and POST definition for each gene. We also implemented an efficient strategy to consider multiple APA sites for every gene, calculating m/M, roar and *p*-values for each one of them in combination with the canonical end of the transcript.

For the analyses presented here we define the canonical transcripts ends using RefSeq annotations from UCSC ([27–29]) and collapsing together the structures of all the transcripts assigned to a gene, defining in the most conservative way exons and UTRs by getting the union of all the exons and defining the 3’ (5’) UTR using the most 5’ distal (proximal) coding end (start). Moreover we kept only mRNA RefSeqs.

We used as alternative ends annotation sources two different databases: APASdb [15] and PolyA_DB [14]. The former is based on an ad-hoc sequencing protocol that sequences the RNA near poly-A tails (SAPAS) followed by reads alignment and clustering to define transcripts ends, filtering out possible false positives derived from internal priming with stretches of adenines on the genomic sequence. The latter is based on similar principles but uses as starting data cDNA/ESTs that contain a stretch of A or T after their aligned portion. APASdb offers data for 22 normal human tissues, some cancer tissues, the murine thymopoiesis, zebrafish embryonic development and some lancelet samples while PolyA_DB for human, mouse, rat, chicken and zebrafish using all the cDNA/EST sequences available in the respective UniGene databases from NCBI.

The choice of the Refseq annotation to define the canonical polyadenylation site instead of one of the sites reported in the APA databases is justified by the fact that in most cases the site reported by APASdb as being supported by most reads falls near the end defined using Refseq. Moreover this procedure can be applied even when datasets do not report information about the number of supporting reads (i.e PolyA_DB) for each site and thus makes results obtained with APASdb and PolyA_DB more easily comparable.

To compute the distance between transcript ends and the most used APASdb alternative site we put together annotations for all the 22 human normal tissues, normalize reads by the total number of reads found in different tissues, sum them and consider the sites supported by more than 2 normalized reads and that overlaps with our genes - the median distance between the “RefSeq end” and the site supported by the highest number of reads for each gene is 9 (see Fig. 1). This small distance suggests that indeed these two cleavage sites refer to the same major site ([30]).

In the single APA version of the algorithm we choose the most distal APA site (with respect to the transcript end) associated to a gene referring only to sites inside the 3’UTR when possible. POST is then defined as the portion of a transcript between the chosen site and the transcript end and PRE as the portion starting with the beginning of the exon that contains the site and the site itself. Limiting the PRE portion to this exon without further extending it towards the transcript start should avoid noise in read counts derived from alternative splicing events involving other transcript portions and also make the approach less prone to suffer from possible 3’ bias in reads distribution. We decided to exclude APA sites found in introns and prefer those in 3’UTRs when available to focus our attention on bona fide alternative polyadenylation events and to avoid difficulties that could arise when computing the lengths of various transcripts portions in the multiple APA version of the software.

For the multiple APA version when we had a single sample for each condition (or a known sample pairing) we selected for every gene the most significant Fisher test *p*-value (or combined *p*-value) and used that as the representative result for that gene in the following analyses. When there multiple samples for both conditions without a pairing between them we resorted to choosing for each gene the result that had a Fisher test nominal *p*-value <0.05 in every pair and solving ties preferring the result with the smallest product of all *p*-values.

**Fig. 2:**
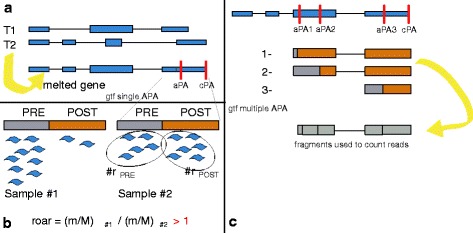
Pipeline. **a** - how we define gene structures starting from different transcripts. We obtain “melted genes” collapsing together the structures of all the transcripts assigned to a gene. aPA: alternative polyadenylation site. cPa: canonical polyadenylation site. Thicker blue rectangles represent coding exons, while the others depict untranslated regions. **b** - an example of how roar works with the single APA annotation: in sample #1 the shorter isoform is more expressed than the longer one with respect to sample #2. Blue wavy shapes represent aligned mRNAseq reads. **c** - how transcript fragments are defined in multiple APA analyses to efficiently count reads for all the possible APA choices. aPA1-2-3 are three different APA sites reported for this sample gene

**Fig. 3:**
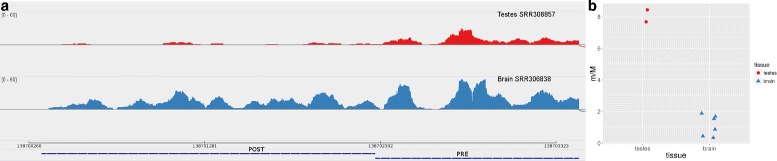
Example of read density and corresponding m/M values. **a**- Sashimi plot produced with IGV of two alignments for representative samples for testes and brain over the PRE and POST portions that we consider for CAMSAP1, one of the genes with the strongest shortening signal in testes versus brain. Read density is clearly lower in testes on the POST portion. CAMSAP1 is on the negative strand and the PRE fragment overlaps with the coding portion and the beginning of the 3’UTR of its last exon. **b**- Dot plot representing the m/M values obtained for the two testes and six brain samples. The larger m/M values for testes reflect the preferential expression of the short isoform in that tissue

Figure 2 briefly depicts our strategies to define PRE and POST portions and the software algorithm at a glance, while Fig. 3 represents one anecdotal example of how read densities over PRE and POST portions are reflected in m/M values - we have chosen one of the genes with the highest roar (9.63, thus shortened in the first condition) from one of the comparisons presented in the “Results”.

### m/M calculations

To correctly evaluate the ratio of read counts, we have to take into account that reads falling over PRE could have been obtained from both isoforms while the ones falling on POST derive exclusively from the longer one; another more trivial question that needs to be addressed is that reads fall with larger frequencies on longer stretches of RNA.

We can say that the total number of reads falling over a transcript in its entirety (*N*) derives from the relative abundance of the two isoforms and their potential to generate reads; that is: *N*=*ε*_*M*_ *M*+*ε*_*m*_ *m* where *m* is the quantity of the short isoform, *M* of the long one and *ε* identifies their efficiency in generating reads.

Assuming the equiprobability of read distribution (that is each nucleotide has the same probability of finding itself in a read) the efficiency in generating reads of the two isoforms is proportional (with a constant *k*) to their lengths:

$$\epsilon_{M} = k (l_{PRE}+l_{POST}) $$

$$\epsilon_{m} = k (l_{PRE}) $$

Defining *l*_*PRE*_ as the length of the PRE portion and *l*_*POST*_ as the length of the POST we can now obtain the number of reads falling on the two portions as:

$$\#r_{PRE} = \epsilon_{M} M \left(\frac{l_{PRE}}{l_{PRE}+l_{POST}}\right) + \epsilon_{m} m $$

and:

$$\#r_{POST} = \epsilon_{M} M \left(\frac{l_{POST}}{l_{PRE}+l_{POST}}\right) $$

These two equations reflect the fact that all the reads deriving from the short isoform (*ε*_*m*_ *m*) fall on the PRE portion while the ones deriving from the long one are distributed over the PRE and POST portions depending on their lengths.

We can now setup a system of equations aimed at obtaining the *m*/*M* value in terms of the numbers of reads falling over the two portions and their lengths.

We start from:

$$\left\{ \begin{array}{lr} \#r_{PRE} = & \epsilon_{M} M \left(\frac{l_{PRE}}{l_{PRE}+l_{POST}}\right) \\[0.3cm] & + \epsilon_{m} m \\[0.25cm] \#r_{POST} = & \epsilon_{M} M \left(\frac{l_{POST}}{l_{PRE}+l_{POST}}\right) \end{array} \right. $$

Then with simple algebraic steps the system can be solved yielding the formula to obtain *m*/*M* using only read counts and lengths:

$$m/M = \frac{l_{POST} \#r_{PRE}}{l_{PRE} \#r_{POST}} -1 $$

As a simple emblematic case suppose that the PRE and POST portions have the same lengths and that the short and long isoforms are in perfect equilibrium (i.e. they are present in a cell in equal numbers). In this situation we will find on the PRE portion two times the number of reads falling on the POST one because half of them will derive from the long isoforms and the other half from the short ones. In this case the equation correctly gives us an *m*/*M* equal to 1.

In the previous discussion we have ignored reads falling across the PRE/POST boundaries. As long as they can derive only from the long isoform it is reasonable to assign them to *#* *r*_*POST*_. To simplify the implementation of this strategy in the multiple APA version of the software we consider reads as falling on a single base at their most 3’ distal end.

Portion lengths should be corrected to keep into account read lengths and the assignment to POST of spanning reads, therefore:

$$l^{\prime}_{PRE} = l_{PRE} $$

$$l^{\prime}_{POST} = l_{POST} + readLength - 1 $$

Normally we should have added readLength to both the lengths but in this case we do not expect reads to fall after the POST portion (that is the end of transcripts) and thus we only have to correct for the spanning reads. We do not subtract the same value from *l*_*PRE*_ as long as in theory we could expect reads to fall at its 5’ (i.e. reads falling across that exon and the previous one or those from still unspliced transcripts). These corrected lengths are those used for the *m*/*M* calculations.

### Bioconductor package

The algorithm is implemented in a Bioconductor package that takes as input bam files with the alignment of the RNA-seq reads coming from two experimental conditions and a gtf file with coordinates of genes and APA sites to be analyzed.

As we previously mentioned, the package could be used in two ways: with a single PRE/POST definition for every gene for which the user provides coordinates and lengths of the PRE and POST portions (on the transcriptome and not on the genome, i.e. excluding introns) or with multiple APA sites for every gene; in the latter case the user must provide the exon structures of the desired genes and the coordinates of the sites that have to be considered. The package will then automatically identify all the possible pairs of PRE and POST portions for the genes (with POST always ending at the end of the given transcripts) and their lengths. Practically the second approach works also with genes harbouring a single APA but we left the first option for ease of use and to avoid breaking the interface of our first version of the Bioconductor package. Internally the management of the multiple APA analyses is performed without having to count the same reads many times for all the PRE/POST choices but more efficiently by counting reads falling on all the relevant portions of the genes and summing the appropriate combinations needed to obtain the different # *r*_*PRE*_ and # *r*_*POST*_ (see Fig. 2 c).

The results between a single and a multiple analysis are not identical even when in principle they should be (i.e. for genes with a single APA or for the same gene-APA pairs) due to programming choices made for efficiency reasons. The first difference is due to the “shrinkage” of reads to a single base at their most 3’ distal end and the fact that we align and count them on distinct fragments of the genes: if a very long read spans several fragments pertaining to different POST portions (for different choices of APA sites of the same gene) in the multiple version it will be counted only as aligning on the most 3’ distal fragment and therefore on a single POST. The second cause of small differences is due to genes with overlapping 3’UTRs: the single version correctly discards reads that align on the overlapping portion and therefore whose transcript of origin is not univocally identifiable. The multiple one manages genes on different strands separately and therefore counts these kind of reads as aligned on all the genes that they overlap (if the genes are on different strands). This second problem in the multiple analyses could be overcome by supplying annotations without overlapping genes. The only way to solve this problem and the first one together in a multiple APA analysis context would be to use the single APA one many times (one for every choice of APA for the genes of interest) - this would be very time consuming and inefficient. The multiple APA version has been implemented for ease of use and efficiency and in spite of the cited details when we compare its results with the single version for one of the datasets presented in the “Results” we get identical roar values in 3870 genes over 7498 and a pearson correlation of 0.82 between them.

We provide ([31]) gtf files for hg19 and mm9 genome releases using PolyA_DB2 or APASdb (only for hg19) as APA annotation sources, for both the single and multiple APA analyses.

The package is well integrated with the Bioconductor infrastructure and presents different methods for each analysis step (obtaining counts, m/M, roar, Fisher test *p*-values), allowing the user to choose filtering parameters (in terms of expression levels, Fisher test *p*-values,...) and to study the results of each phase of the pipeline.

## Results

### Validation of the approach

To validate our procedure we compared its results with two completely different methodologies: a microarray based one [11] and one that uses an ad hoc deep sequencing library extraction [13].

For the first comparison we used human RNAseq data obtained from brain and testes [32, 33], where we expect to find a notable preference towards shorter isoforms in testes: this is indeed what we found (205 shortened genes in testes versus brain, and only 7 lengthened). The overlap between the 845 genes found shortened (and 56 lengthened) with the microarray based approach (using data from [34–40], see Additional file 1 for the complete samples lists) and ours was significant (104 common genes, Fisher test *p*-value 4.41×10^−48^). For lengthened genes the result is not significant due do the limited number of involved genes.

**Fig. 4:**
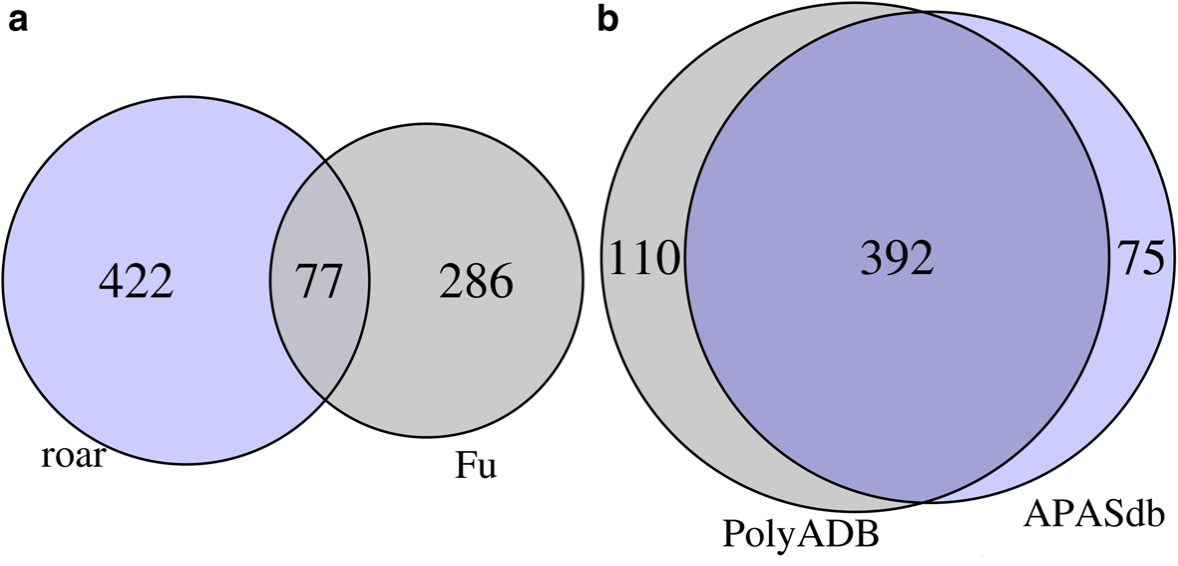
Venn diagrams of overlaps between roar results and a standard approach and between two different annotations for roar. **a** - MCF7 vs MCF10: overlap between shortened genes for roar and [13] **b** - MCF7 vs MCF10: overlap between shortened genes for roar using PolyA_DB or APASdb

The second comparison focused on human breast cancer and normal tissue cell lines where we performed 3 comparisons (MCF7 vs MCF10, MDA-MB231 vs MCF10, MDA-MB231 vs MCF7) as in [13] but on mRNA-seq data ([41, 42]): in all cases but one we obtained significant overlaps (*p*-values 5.4×10^−21^,8×10^−12^,0.0001 for shortened genes and 0.12,0.0023,0.00035 for lengthened ones, Fig. 4 a).

These results were obtained with APASdb derived annotations choosing for every gene the APA site that determines the most extreme shortening effect, favouring those inside the 3’UTR when available. We observed almost completely superimposable (Fig. 4 b and Additional file 2: Table S1) results when using PolyA_DB with single sites.

When using either database with multiple APA sites for every gene as expected we found practically all the genes of the single APA analyses plus some other genes, this resulted on average in moderately better overlaps with other approaches (1.72×10^−59^ in testes vs brain for shortened genes and 1.08×10^−35^,2.19×10^−12^,0.001 for shortened and 0.009,0.009,2.51×10^−10^ for lengthened genes in breast cell lines. See Additional file 3: Table S2 for the results obtained using PolyA_DB).

### Comparisons with DaPars

We then compared our results using APASdb with a single APA choice on the same dataset used in [16] with DaPars: roar detects 664 (1) of the 818 (1) genes found shortened (lengthened) by DaPars in the CFIm25 knockdown cells and moreover finds 1136 (39) other genes with a significant signal towards shortening (lengthening); this suggests that indeed exploiting previous knowledge about alternative polyadenylation sites provides increased statistical power in detecting alternative polyadenylation events. For this comparison to have the same type I error we used the DaPars results reported by the article with its standard cutoffs, in particular with a FDR ≤0.05; for roar having two samples for each condition without any known pairing we decided to be very conservative and use for every gene the highest *p*-value of all the four possible pairings, correct it with Bonferroni and then use a 0.05 cutoff.

To further delve into this issue we ran DaPars and roar on a random subset of the reads (1/15 of the total mapped reads, reaching a quantity of reads more similar to the suggested one ([43]) for differential expression analyses rather than for studies on transcripts structure): roar still detects 86 (0) of the 120 (0) genes found shortened (lengthened) by DaPars but moreover detects 487 (3) other genes (with results significantly overlapping with those obtained on the whole dataset: Fisher *p*-value <1×10^−30^ for shortening and 3.03×10^−7^ for lengthening). We also ran DaPars on the two datasets used to validate roar but with the default cutoffs it did not detect any significant shortening or lengthening.

These results confirm that our approach is indeed able to work on datasets obtained for standard differential expression analyses without the need for higher read depth.

## Discussion

Comparisons with other approaches yielded significant overlaps but also a non-negligible number of genes where alternative polyadenylation sites usage was detected only by our approach or by the other one. Considering for example Fig. 4 we can identify different possible causes for the genes independently detected by the two strategies. From a technical point of view Fu et al. [13] used a completely different approach with some filters on genes (for example considering only cleavage sites found in the last exon) that could explain at least partially the 422 genes found only by roar. Moreover mRNAseq and SAPAS could have slightly different biases towards different genes - in their Supplementary Material Fu et al. [13] indeed show that the correlation between RNAseq and SAPAS expression values is significant but not perfect and in addition to this the correlation between SAPAS results obtained with Illumina or 454 sequencing is significant but weak, underlining how technical issues could be a source of differences. There could also be a biological source of variation, as long as cancer cell line heterogeneity both from a genetic and phenotypic (i.e. expression levels, [44]) point of view is a known issue and the data used for our comparisons comes from two sets of independent clones from different laboratories. To further investigate the sources of differences we asked ourselves if there are some differences in terms of expression levels between the four sets of genes (detected as shortened by both approaches, only by roar, only by SAPAS or not significant - see Additional file 4: Figure S1) and this is indeed the case: genes identified as shortened only by Fu et al. [13] are less expressed than those (Mann-Whitney U test *p*-value 0.0012) found only by roar or by both approaches. This is not surprising as long as roar statistical power is limited by the number of reads obtained on 3’UTR while the SAPAS approach is less sensitive to this problem. There is one last issue that should always be considered when considering overlaps of methodologies that involve a cutoff on a *p*-value to identify significant results: controlling for false positives inevitably leads to false negatives whose prevalence is not easily controlled, and thus we always expect the overlap between results obtained with different statistical approaches to be partial. Similar technical and biological mechanisms could be behind the testes-brain comparison but the significance of the overlap supports the validity of using roar as a cost effective first line tool.

Limiting analyses to a single APA for every gene instead of considering all the reported APA sites reduces the computational burden of the study (on the testes vs brain dataset the multiple analysis is 10 times slower than the single APA one) but gives a slight disadvantage in terms of overlaps with other shortening detection methods - we decided to offer both possibilities because in the perspective of a “first line” analysis tool in many cases choosing a single APA will be sufficient. Nonetheless we believe that the possibility to efficiently analyse all the reported APA for genes is useful, especially for genes with long 3’UTRs. We decided to separately obtain *m*/*M* and roar values for each APA choice assigning reads every time either to a single PRE or POST portion to avoid over-complicating our model and falling back to the complex issue of transcript structure inference - choosing the most significant Fisher test is sufficient to focus one’s attention on the most robust signal to detect shortening.

One last point that could foster further work in future releases of the package is related to library depth normalization: the algorithm is based on the Fisher test to detect significant results - this is an exact test that does not need balancing between the columns of its 2x2 tables therefore our results are robust from this point of view. The question about *m*/*M* and roar calculation is slightly more complex: for comparisons without replicates we work on two *m*/*M* values that derive from ratios of read counts in the samples being compared therefore we do not need to apply a correction on library depth. When there are replicates we perform reads counts averages therefore normalization could be an issue but it is not straightforward to tackle because simply correcting using total read counts is considered outdated [45]. Trying to understand how to correct *m*/*M* values for multiple samples settings with high library depth imbalance is an interesting problem that could be addressed in a future release of the roar package.

## Conclusion

Our tool can be a useful component of the arsenal for first line analyses of RNAseq experiments because it is able to detect whether alternative polyadenylation is a phenomenon that is relevant in the comparison of two different biological conditions without needing a specific experimental setup or the read depth usually required for the analysis of alternative isoforms. A crucial element that makes this possible is the use of polyadenylation databases as an annotation source, which greatly reduces the required depth compared to methods which attempt to determine the APA sites directly from the data and allows instead the use of datasets of the size typically used for differential expression analysis. The results of roar could then be used to decide whether to refine the experimental and computational investigations in order to study alternative polyadenylation in a more complete way, possibly involving the detection of new, unannotated APA sites.

## Availability and requirements

- **Project name**: roar
- **Project home page**: http://bioconductor.org/packages/release/bioc/html/roar.html,
https://github.com/vodkatad/roar/
- **Operating systems**: any operating system supporting R
- **Programming language**: R
- **Other requirements**: working R and Bioconductor installation
- **Licence**: GNU GPL-3
- **Any restriction to use by non-academics**: none

## Endnote

^1^ The multiple test correction takes place for the genes that are considered expressed and with a defined value of roar.

## Additional files

Additional file 1 Lists of samples (GSE and GSM identifiers) with microarray data for human brain and testes. (TXT 5 kb)

Additional file 2 Concordant results using different annotation sources and single or multiple APA analyses Spreadsheet reporting the genes reported as shortened or lengthened with roar using either the APASdb or PolyA_DB annotation with the single or multiple APA analyses and overlaps between the lists. (ZIP 7 kb)

Additional file 3 Multiple APA analyses results with PolyA_DB: comparisons with other approaches. (XLS 8 kb)

Additional file 4 MCF7/MCF10 comparisons vs Fu - density plots of FPKM _*PRE*_ values for different classes of genes: “Common” are genes detected as shortened by both approaches, “Only roar” and “Only Fu” are genes identified by only either one of the approaches, “N.S.” are genes not significantly identified as shortened. FPKM _*PRE*_ values are averaged between the MCF7 and MCF10 samples. (PDF 456 kb)

## Abbreviations

APA: Alternative polyadenylation
FPKM: Fragments per kilobase of transcript per million mapped reads
UTR: Untranslated region
