# Supplementary figures and images for: Roar: detecting alternative polyadenylation with standard mRNA sequencing libraries
Source: BMC Bioinformatics. 2016 Oct 18;17:423. doi: 10.1186/s12859-016-1254-8 (PMC5069797; doi:10.1186/s12859-016-1254-8)

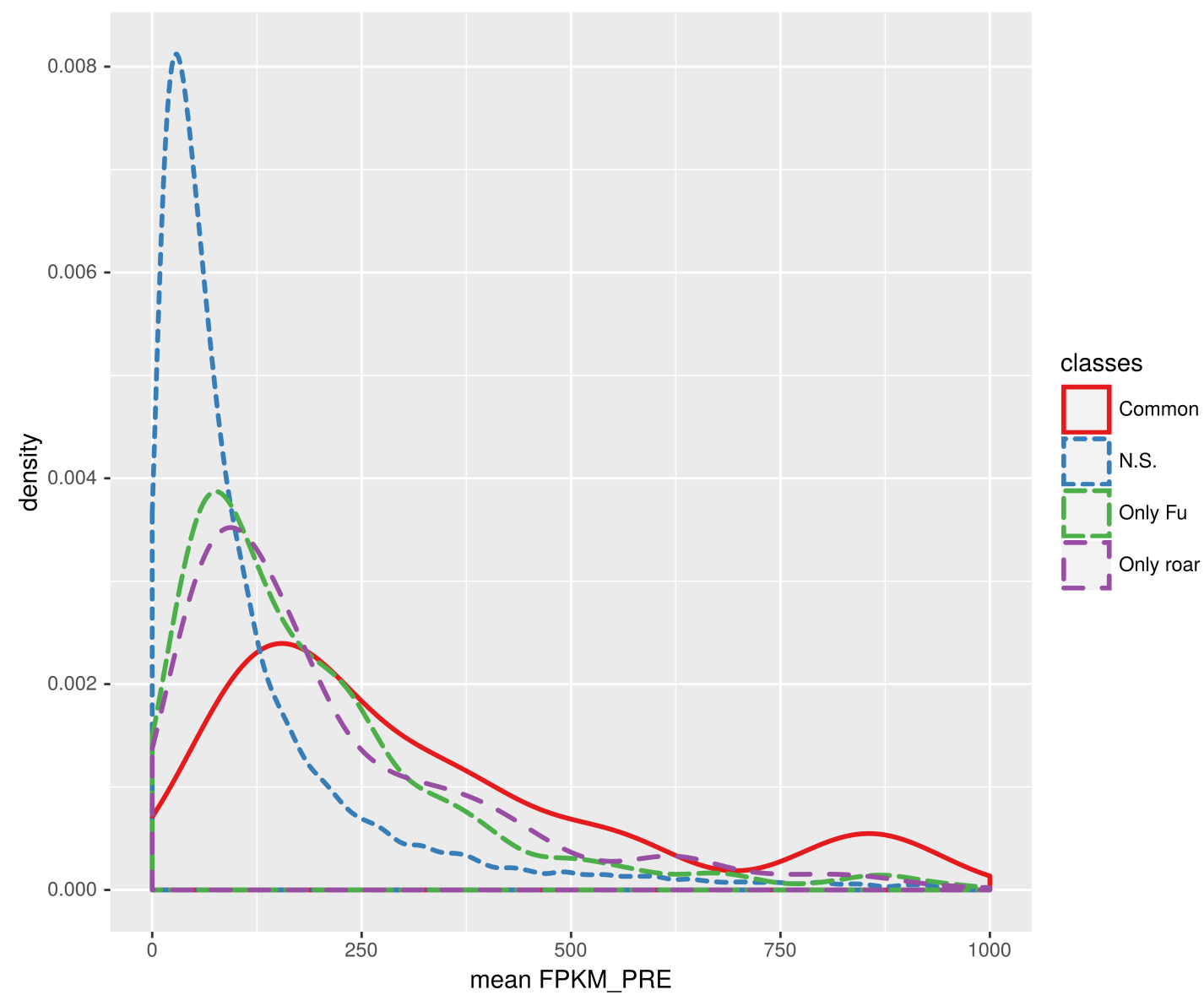

Supplement: Additional file 4 — MCF7/MCF10 comparisons vs Fu - density plots of FPKM PRE values for different classes of genes: “Common” are genes detected as shortened by both approaches, “Only roar” and “Only Fu” are genes identified by only either one of the approaches, “N.S.” are genes not significantly identified as shortened. FPKM PRE values are averaged between the MCF7 and MCF10 samples. (PDF 456 kb) [file 12859_2016_1254_MOESM4_ESM.pdf]
